# Supplementary material for: Epidemiology of Cerebral Palsy among Children and Adolescents in Arabic-Speaking Countries: A Systematic Review and Meta-Analysis
Source: Brain Sci. 2022 Jun 29;12(7):859. doi: 10.3390/brainsci12070859 (PMC9313288; doi:10.3390/brainsci12070859)
Supplement: Supplementary file 1 [file brainsci-12-00859-s001.zip › brainsci-1761018-supplementary.pdf]

Supplementary: Medline Search

Systematic review search strategy

Database: Ovid MEDLINE(R) ALL <1946 to July 09, 2021>
Search Strategy:
----
1 exp Cerebral Palsy/
2 (cerebral adj pals$).tw.
3 CP.tw.
4 1 or 2 or 3
5 exp Epidemiology/
6 epidemiolog$.tw.
7 exp "Cost of Illness"/
8 burden$.tw.
9 exp Hospitalization/
10 exp Morbidity/
11 exp Mortality/
12 exp Death/
13 (hospital$ or morbid$ or mortalit$ or death$).tw.
14 exp Incidence/
15 exp Prevalence/
16 (inciden$ or prevalen$).tw.
17 exp Disease Notification/
18 (notif$ or case$).tw.
19 (sign$ or symptom$ or feature$ or manifest$ or present$ or finding$ or characteristic$ or criteri$).tw.
20 exp Diagnosis/
21 diagnos$.tw.
22 exp Prognosis/
23 prognos$.tw.
24 (aetiolog$ or etiolog$).tw.
25 5 or 6 or 7 or 8 or 9 or 10 or 11 or 12 or 13 or 14 or 15 or 16 or 17 or 18 or 19 or 20 or 21 or 22 or 23 or 24
26 4 and 25
27 exp Jordan/
28 jordan$.tw.
29 exp United Arab Emirates/

30 emirate$.tw.
31 uae.tw.
32 exp Bahrain/
33 bahrain$.tw.
34 exp Tunisia/
35 tunisia$.tw.
36 exp Algeria/
37 algeria$.tw.
38 exp Djibouti/
39 djibouti$.tw.
40 exp Saudi Arabia/
41 (saudi adj1 arabia$).tw.
42 exp Sudan/
43 sudan$.tw.
44 exp Syria/
45 syria$.tw.
46 exp Somalia/
47 somalia$.tw.
48 exp Iraq/
49 iraq$.tw.
50 exp Oman/
51 oman$.tw.
52 palestin$.tw.
53 exp Qatar/
54 qatar$.tw.
55 exp Comoros/
56 comoros$.tw.
57 exp Kuwait/
58 kuwait$.tw.
59 exp Lebanon/
60 leban$.tw.
61 exp Libya/
62 libya$.tw.
63 exp Egypt/
64 egypt$.tw.
65 exp Morocco/
66 morocco$.tw.

67 exp Mauritania/
68 mauritania$.tw.
69 exp Yemen/
70 yemen$.tw.
71 exp Arabs/
72 (arab$ adj4 (speak$ or countr$ or world)).tw.
73 27 or 28 or 29 or 30 or 31 or 32 or 33 or 34 or 35 or 36 or 37 or 38 or 39 or 40 or 41 or 42 or 43 or 44 or 45 or 46 or 47 or 48 or 49 or 50 or 51 or 52 or 53 or 54 or 55 or 56 or 57 or 58 or 59 or 60 or 61 or 62 or 63 or 64 or 65 or 66 or 67 or 68 or 69 or 70 or 71 or 72
74 26 and 73
75 limit 74 to "all child (0 to 18 years)"
76 exp Infant, Newborn/
77 exp Infant/
78 exp Child, Preschool/
79 exp Child/
80 exp Adolescent/
81 (baby or babies or infant$ or toddler$ or child$ or adolescen$ or pediatric$ or paediatric$).tw.
82 76 or 77 or 78 or 79 or 80 or 81
83 74 and 82
84 75 or 83

Table S1. Risk of bias (ROB) assessment and quality scores for included studies.

First author, year	Country [1]	Design	Tool	Selection/ Ascertainment	Comparability/ Causality	Outcome/ Exposure/ Reporting
Abas et al., 2017 [1]	Egypt	Cross-sectional	NOS for cross-sectional	★★	★	★★
El-Tallawy et al., 2011 [2]	Egypt	Cross-sectional	NOS for cross-sectional	★★★	★	★★★
El-Tallawy et al., 2014 [3]	Egypt	Case-control	NOS for case-control and cohort	★★★	★★	★★
El-Tallawy et al., 2014 [4]	Egypt	Cross-sectional	NOS for cross-sectional	★★★	★★	★★
Reyad et al., 2017 [5]	Egypt	Cross-sectional	NOS for cross-sectional	★★★	NA	★★
Yasser et al., 2016 [6]	Egypt	Cross-sectional	NOS for cross-sectional	★★★★	NA	★★
Hassan et al, 2009 [7]	Iraq	Case-series	Assessment tool for risk of bias case series	★★	-	★
Kareem et al., 2009 [8]	Iraq	Case-series	Assessment tool for risk of bias case series	★★★	★	★
Khadir et al., 2020 [9]	Iraq	Case-series	Assessment tool for risk of bias case series	★★	-	-

Salman et al., 2019 [10]	Iraq	Case-series	Assessment tool for risk of bias case series	★★	★	★
Al Ajlouni et al., 2006 [11]	Jordan	Case-series	Assessment tool for risk of bias case series	★★	-	★★
Al-Ajlouni et al, 2008 [12]	Jordan	Case-series	Assessment tool for risk of bias case series	★★★	-	★
Almasri et al., 2018 [13]	Jordan	Case-series	Assessment tool for risk of bias case series	★★★	★	★
Almasri et al., 2018 [14]	Jordan	Case-series	Assessment tool for risk of bias case series	★★	★	★
Almasri et al., 2019 [15]	Jordan	Cross-sectional	NOS for cross-sectional	★★	NA	★★★
Janson et al., 1994 [16]	Jordan	Cross-sectional	NOS for cross-sectional	★★★★	★	★
Nafi et al., 2012 [17]	Jordan	Case-series	Assessment tool for risk of bias case series	★★	★	★
Saleh et al., 2013 [18]	Jordan	Case-series	Assessment tool for risk of bias case series	★★	★	★
Khan et al., 1992 [19]	Libya	Case-control	NOS for case-control and cohort	★★	★★	★
Daher et al., 2014 [20]	Palestine	Case-control	NOS for case-control and cohort	★★★★	★	★★★

Abolfotouh et al., 2018 [21]	Saudi Arabia	Cohort	NOS for cohort	★★★★	★	★★★
Al Salloum et al., 2011 [22]	Saudi Arabia	Cross-sectional	NOS for cross-sectional	★★★	-	★★
Al-Asmari et al., 2006 [23]	Saudi Arabia	Case-series	Assessment tool for risk of bias case series	★★★	-	★
Alfrayh et al., 1987 [24]	Saudi Arabia	Case-series	Assessment tool for risk of bias case series	★★★	★	★
Almuneef et al., 2019 [25]	Saudi Arabia	Cross-sectional	NOS for cross-sectional	★★	★	★★
Al-Naquib et al., 1988 [26]	Saudi Arabia	Case-series	Assessment tool for risk of bias case series	★★	★	★
Al-Rajeh et al., 1991 [27]	Saudi Arabia	Case-control	NOS for case-control and cohort	★★	-	★
Al-Sulaiman et al., 2003 [28]	Saudi Arabia	Case-series	Assessment tool for risk of bias case series	★★★	-	★
Izuora et al., 1992 [29]	Saudi Arabia	Case-series	Assessment tool for risk of bias case series	★★	★	★
Taha et al., 1984 [30]	Saudi Arabia	Case-series	Assessment tool for risk of bias case series	★★★	★	★
Abdullahi et al., 2013 [31]	Sudan	Case-control	NOS for case-control and cohort	★★★	★★	★★

Salih et al., 2020 [32]	Sudan	Case-series	Assessment tool for risk of bias case series	★★	★★	★

[1] Ordered alphabetically by country name. NA: not applicable.

Studies were excluded due to unacceptably high risk of bias with additional data regarding their risk of bias and exclusion:

- An overlap between participants in the study (Al-Sulaiman A., 2003) [28] and those reported in the other studies (El Rifai M., 1984 and El Rifai M., 1984) [33,34]. Both include the same study population. The recent study (Al-Sulaiman A., 2003) [28] has been included only.
- The risk factors and motor disabilities in the study (Al-Wazna T., 1997) [35] don't seem to be reported specifically for people with CP, but for the whole group including people with other disabilities.
- Study (Al-Sulaiman A., 1997) [36]: This study lacked a clear definition of some terms and displayed some discrepancies in number totals.
- Studies (El-Tallawy H., 2014 and El Tallawy H., 2013) [4,37] were on the same population and almost the same authors. We only included one study [4] that was mainly discussing CP.
- Study (El Tallawy H., 2010) [38] was on the same population that in study (El-Tallawy H., 2011) [2]. Therefore, we included the recently published one (i.e., El-Tallawy H., 2011) [2].
- Studies (Al Rajeh S., 1993 and Al-Turaiki M., 1994) [39,40] were excluded as they did not meet the inclusion criteria for age.
- Studies (Saleh M., 2017 and Almasri N., 2019) [15,41] both were on the same study population. We only included the most recent one [15].

Bibliography:

1. Abas, O.; Abdelaziem, F.; Kilany, A. Clinical spectrum of cerebral palsy and associated disability in South Egypt: A local survey study. *Macedonian Journal of Medical Sciences* **2017**, *5*, 37-41, doi:10.3889/oamjms.2017.020.
2. El-Tallawy, H.N.; Farghaly, W.M.A.; Shehata, G.A.; Metwally, N.A.; Rageh, T.A.; Abo-Elfetoh, N. Epidemiology of cerebral palsy in El-Kharga District-New Valley (Egypt). *Brain and Development* **2011**, *33*, 406-411, doi:10.1016/j.braindev.2010.07.011.
3. El-Tallawy, H.N.; Farghaly, W.M.A.; Shehata, G.A.; Badry, R.; Rageh, T.A. Epileptic and cognitive changes in children with cerebral palsy: An Egyptian study. *Neuropsychiatric Disease and Treatment* **2014**, *10*, 971-975, doi:10.2147/NDT.S59600.
4. El-Tallawy, H.N.; Farghaly, W.M.A.; Shehata, G.A.; Rageh, T.A.; Metwally, N.A.; Badry, R.; Sayed, M.A.M.; El Hamed, M.A.; Abd-Elwarth, A.; Kandil, M.R. Cerebral palsy in Al-Quseir City, Egypt: Prevalence, subtypes, and risk factors. *Neuropsychiatric Disease and Treatment* **2014**, *10*, 1267-1272, doi:10.2147/NDT.S59599.

5. Reyad, A.; Abdelaziem, F.H.; Kilany, A. Physical Therapy Registry for Establishment of Cerebral Palsy in Alexandria City (Almontazah District), Egypt. *IOSR Journal of Nursing and health Science* **2017**, *06*, 20-24.
6. Yasser, S.; FatenAbdelaziem; Eltallawy, H. Establish registry of cerebral palsy in mit-ghamer city , Egypt. 2016.
7. Hassan, K.H. Cerebral palsy among Kurdish children in the city of Dohuk: A case-series study. *Jordan Medical Journal* **2009**, *43*, 205-211.
8. Kareem, A.A.; Kamel, M.A.S. Risk factors and clinical profiles in Iraqi children with cerebral palsy. *New Iraqi Journal of Medicine* **2009**, *5*, 64-68.
9. Khadir, S.; Issa, S.A. Magnetic resonance imaging findings in patients with cerebral palsy in Duhok, Iraq: Case series. *Journal of Surgery & Medicine (JOSAM)* **2020**, *4*, 1-4, doi:10.28982/josam.663221.
10. Ahmed, A.; Salman; Arab, M.N.; Sarhat, A.R. Cerebral palsy epidemiology in Tikrit-Iraq. *Indian Journal of Public Health Research and Development* **2019**, *10*, 942-947, doi:10.5958/0976-5506.2019.00182.7.
11. Al Ajlouni, S.F.; Aqrabawi, M.; Oweis, N.; Daoud, A.S. Clinical spectrum of cerebral palsy in Jordanian children: An analysis of 200 cases. *Journal of Pediatric Neurology* **2006**, *4*, 251-255, doi:10.1055/s-0035-1557333.
12. Al-Ajlouni, S.; Alagrabawi, M.; Al-Balas, H.; Alawneh, M.; Daoud, A. Cerebral palsy in Jordan: Clinical and neuroimaging characteristics. *Jordan Medical Journal* **2008**, *42*, 162-169.
13. Almasri, N.A.; Saleh, M.; Abu-Dahab, S.; Malkawi, S.H.; Nordmark, E. Development of a Cerebral Palsy Follow-up Registry in Jordan (CPUP-Jordan). *Child: Care, Health & Development* **2018**, *44*, 131-139, doi:10.1111/cch.12527.
14. Almasri, N.A.; Saleh, M.; Abu-Dahab, S.; Malkawi, S.H.; Nordmark, E. Functional profiles of children with cerebral palsy in Jordan based on the association between gross motor function and manual ability. *BMC Pediatrics* **2018**, *18*, N.PAG-N.PAG, doi:10.1186/s12887-018-1257-x.
15. Almasri, N.A.; Dunst, C.J.; Saleh, M.; Okasheh, R. Determinants of Utilization of Health Services Provided for Children with Cerebral Palsy in Jordan. *Journal of Developmental & Physical Disabilities* **2019**, *31*, 205-217, doi:10.1007/s10882-018-9629-6.
16. Janson, S.; Dawani, H. Chronic illness in preschool Jordanian children. *Annals of Tropical Paediatrics* **1994**, *14*, 137-144, doi:10.1080/02724936.1994.11747706.
17. Nafi, O.A. Clinical spectrum of cerebral palsy in South Jordan: Analysis of 122 cases. *Jordan Medical Journal* **2012**, *46*, 210-215.
18. Saleh, M.; Almasri, N.A. Use of the Measure of Processes of Care (MPOC-20) to evaluate health service delivery for children with cerebral palsy and their families in Jordan: validation of Arabic-translated version (AR- MPOC-20). *Child: Care, Health & Development* **2014**, *40*, 680-688, doi:10.1111/cch.12116.
19. Khan, M.A. Intellectual and developmental assessment of cerebral palsy cases in Libyan city. *Indian journal of medical sciences* **1992**, *46*, 235-238.
20. Daher, S.; El-Khairy, L. Association of cerebral palsy with consanguineous parents and other risk factors in a Palestinian population. *Eastern Mediterranean Health Journal* **2014**, *20*, 459-468, doi:10.26719/2014.20.7.459.
21. Abolfotouh, M.A.; Al Saif, S.; Altwaijri, W.A.; Al Rowaily, M.A. Prospective study of early and late outcomes of extremely low birthweight in Central Saudi Arabia. *BMC Pediatrics* **2018**, *18*, N.PAG-N.PAG, doi:10.1186/s12887-018-1248-y.
22. Al Salloum, A.A.; El Mouzan, M.I.; Al Omar, A.A.; Al Herbish, A.S.; Qurashi, M.M. The prevalence of neurological disorders in saudi children: A community-based study. *Journal of Child Neurology* **2011**, *26*, 21-24, doi:10.1177/0883073810371510.

23. Al-Asmari, A.; Al Moutaery, K.; Akhdar, F.; Al Jadid, M. Cerebral palsy: incidence and clinical features in Saudi Arabia. *Disability & Rehabilitation* **2006**, *28*, 1373-1377, doi:10.1080/09638280600638083.
24. Alfrayh, A.; Naquib, N.A. The pattern of central nervous disease in children in king khalid university hospital in Riyadh, Saudi Arabia. *Journal of Tropical Pediatrics* **1987**, *33*, 124-130, doi:10.1093/tropej/33.3.124.
25. Almuneef, A.R.; Almajwal, A.; Alam, I.; Abulmeaty, M.; Bader, B.A.; Badr, M.F.; Almuammar, M.; Razak, S. Malnutrition is common in children with cerebral palsy in Saudi Arabia - a cross-sectional clinical observational study. *BMC Neurology* **2019**, *19*, 1-10, doi:10.1186/s12883-019-1553-6.
26. Al-naquib, N. Neuro-developmental problems in children in riyadh, saudi arabia: 1-year's experience in a family practice centre. *Journal of Tropical Pediatrics* **1988**, *34*, 294-300, doi:10.1093/tropej/34.6.294.
27. Al-Rajeh, S.; Bademosi, O.; Awada, A.; Ismail, H.; Al-Shammasi, S.; Dawodu, A. CEREBRAL PALSY IN SAUDI ARABIA: A CASE-CONTROL STUDY OF RISK FACTORS. *Developmental Medicine & Child Neurology* **1991**, *33*, 1048-1052, doi:10.1111/j.1469-8749.1991.tb14826.x.
28. Al-Sulaiman, A.A.; Bademosi, O.F.; Ismail, H.M.; Al-Quliti, K.W.; Al-Shammary, S.F.; Abumadini, M.S.; Al-Umran, K.U.; Magbool, G.M. Cerebral palsy in Saudi children. *Neurosciences* **2003**, *8*, 26-29.
29. Izuora, G.I.; Anis, A.S. Neurological disorders in Saudi children at the Al-Majardah General Hospital, Asir Region. *Annals of Saudi Medicine* **1992**, *12*, 191-195, doi:10.5144/0256-4947.1992.191.
30. Taha, S.A.; Mahdi, A.H. Cerebral palsy in Saudi Arabia: A clinical study of 102 cases. *Annals of Tropical Paediatrics* **1984**, *4*, 155-158, doi:10.1080/02724936.1984.11748326.
31. Abdullahi, H.; Satti, M.; Rayis, D.A.; Imam, A.M.; Adam, I. Intra-partum fever and cerebral palsy in Khartoum, Sudan. *BMC Research Notes* **2013**, *6*, doi:10.1186/1756-0500-6-163.
32. Salih, K. Pattern of Cerebral Palsy Among Sudanese Children Less Than 15 Years of Age. *Cureus* **2020**, *12*, e7232.
33. El Rifai, M.R.; Ramia, S.; Moore, V. Cerebral palsy in Riyadh, Saudi Arabia: I. Aetiological factors. *Annals of Tropical Paediatrics* **1984**, *4*, 7-12, doi:10.1080/02724936.1984.11748298.
34. El Rifai, M.R.; Ramia, S.; Moore, V. Cerebral palsy in Riyadh, Saudi Arabia: II. Associations between gestational age, birthweight and cerebral palsy. *Annals of Tropical Paediatrics* **1984**, *4*, 13-17, doi:10.1080/02724936.1984.11748299.
35. Al-Wazna, T.; Bamgboye, E.A. Characteristics of institutionalized disabled in Saudi Arabia. *Saudi Medical Journal* **1997**, *18*, 70-73.
36. Al-Sulaiman, A.A. Neurological disorders in institutionalized patients in the Eastern Province of Saudi Arabia. *Saudi Medical Journal* **1997**, *18*, 387-389.
37. El Tallawy, H.N.A.; Farghaly, W.M.A.; Rageh, T.A.; Shehata, G.A.; Badry, R.; Metwally, N.A.; El Moselhy, E.A.; Hassan, M.; Sayed, M.A.; Waris, A.A.; et al. Door-to-door survey of major neurological disorders (project) in Al Quseir City, Red Sea Governorate, Egypt. *Neuropsychiatric Disease and Treatment* **2013**, *9*, 767-771, doi:10.2147/NDT.S36956.
38. El Tallawy, H.N.A.; Farghaly, W.M.A.; Rageh, T.A.; Shehata, G.A.; Metwaly, N.A.; Elftoh, N.A.; Hegazy, A.M.; El Moselhy, E.A.; Rayan, I.; Al Fawal, B.M.A.; et al. Epidemiology of major neurological disorders project in Al Kharga District, New Valley, Egypt. *Neuroepidemiology* **2010**, *35*, 291-297, doi:10.1159/000320240.
39. Al Rajeh, S.; Bademosi, O.; Ismail, H.; Awada, A.; Dawodu, A.; Al-Freihi, H.; Assuhaimi, S.; Borollosi, M.; Al-Shammasi, S. A community survey of neurological disorders in Saudi Arabia: The thugbah study. *Neuroepidemiology* **1993**, *12*, 164-178, doi:10.1159/000110316.

40. Al-Tunaiki, M.H.; Al-Falahi, L.A. Specialized seating program in riyadh. *Journal of Prosthetics and Orthotics* **1994**, *6*, 52-56, doi:10.1097/00008526-199400620-00006.
41. Saleh, M.; Almasri, N.A. Cerebral palsy in Jordan: Demographics, medical characteristics, and access to services. *Children's Health Care* **2017**, *46*, 49-65, doi:10.1080/02739615.2015.1124770.
